# Body Composition, Inflammation, and 5-Year Outcomes in Colon Cancer

**DOI:** 10.1001/jamanetworkopen.2021.15274

**Published:** 2021-08-30

**Authors:** Christina A. Fleming, Emer P. O’Connell, Richard G. Kavanagh, Donal P. O’Leary, Maria Twomey, Mark A. Corrigan, Jiang H. Wang, Michael M. Maher, Owen J. O’Connor, Henry P. Redmond

**Affiliations:** 1 Surguvant Research Centre, Cork University Hospital, Cork, Ireland; 2 Department of Colorectal Surgery, Cork University Hospital, Cork, Ireland; 3 Department of Radiology, Cork University Hospital, Cork, Ireland

## Abstract

**Importance:**

Obesity, particularly visceral obesity and sarcopenia, are poor prognostic indicators in colon cancer.

**Objectives:**

To explore the association between body composition profiles and 5-year colon cancer outcomes and delineate the associated underlying inflammatory processes.

**Design, Setting, and Participants:**

This multicenter translational cohort study included patients with nonmetastatic colon cancer who did not have underlying chronic inflammatory disorders and were not receiving anti-inflammatory drugs referred to tertiary cancer centers from 2009 to 2015. Preoperative acute phase proteins (white cell count, C-reactive protein, and albumin), cytokines (interleukin [IL]-1b, IL-2, IL-6, IL-10, interferon γ, and tumor necrosis factor α), vascular endothelial growth factor (VEGF), and cell surface receptor expression levels (CD11b and CD14) were measured. All patients underwent follow-up for at least 5 years. Data were analyzed in December 2020.

**Exposure:**

Nonmetastatic colon cancer.

**Main Outcomes and Measures:**

The associations of body composition profiles with 5-year cancer recurrence and disease-specific mortality were analyzed using Mantel Cox log-rank test and Kaplan-Meier curves.

**Results:**

A total of 28 patients were included (median [interquartile range] age, 67 [58-72] years; 22 [78.6%] men). Low skeletal muscle area (SMA) and high visceral to total fat ratio were associated with poor clinical and oncological outcomes, including increased 5-year recurrence (low SMA: hazard ratio [HR], 2.30 [95% CI, 1.41-2.89]; *P* = .04; high visceral to total fat ratio: HR, 5.78 [95% CI, 3.66-7.95]; *P* = .02). High visceral to total fat ratio was associated with increased 5-year disease-specific mortality (HR, 5.92 [95% CI, 4.04-8.00]; *P* = .02). Patients with low SMA who developed a cancer recurrence, compared with those who did not, had higher C-reactive protein (mean [SD], 31.24 [6.95] mg/dL vs 8.11 [0.58] mg/dL; *P* = .003), IL-6 (mean [SD], 1.93 [1.16] ng/mL vs 0.88 [0.14] ng/mL; *P* = .004), VEGF (mean [SD], 310.03 [122.66] ng/mL vs 176.12 [22.94] ng/mL; *P* = .007), and CD14 (mean [SD], 521.23 [302.02] ng/mL vs 322.07 [98.35] ng/mL; *P* = .03) expression and lower albumin (mean [SD], 3.8 [0.6] g/dL vs 43.50 [3.69] g/dL; *P* = .01), IL-2 (mean [SD], 0.45 [0.25] ng/mL vs 0.94 [0.43] ng/mL; *P* < .001), IL-10 (mean [SD], 8.15 [1.09] ng/mL vs 16.32 [4.43] ng/mL; *P* = .004), and interferon γ (mean [SD], 2.61 [1.36] ng/mL vs 14.87 [3.43] ng/mL; *P* = .02) levels. Patients with high visceral to total fat ratio who developed recurrence had higher levels of IL-6 (mean [SD], 5.26 [7.05] ng/mL vs 2.76 [3.11] ng/mL; *P* = .03) and tumor necrosis factor α (mean [SD], 5.74 [4.53] ng/mL vs 4.50 [1.99] ng/mL; *P* = .03).

**Conclusions and Relevance:**

These findings suggest that low SMA and high visceral to total fat ratio were associated with worse colon cancer outcomes and with increased expression of proinflammatory cytokines and VEGF and inhibition of anti-inflammatory cytokines.

## Key Points

**Question:**

Are underlying inflammatory pathways and body composition profiles associated with less favorable 5-year colon cancer outcomes?

**Findings:**

In this cohort study including 28 patients with nonmetastatic colon cancer, high visceral fat and low muscle mass were associated with increased inflammatory activity and poor 5-year cancer outcomes, including recurrence and disease-specific mortality. Low skeletal muscle area and high visceral to total fat ratio were associated with increased expression of proinflammatory cytokines and vascular endothelial growth factor and inhibition of protective inhibitory cytokines in patients with less favorable 5-year cancer outcomes.

**Meaning:**

These findings suggest that less favorable colon cancer outcomes associated with low skeletal muscle area and high visceral to total fat ratio were also associated with increased expression of proinflammatory cytokines and vascular endothelial growth factor and lower levels of protective inhibitory cytokines.

## Teaser

This cohort study examines associations among body composition profiles, inflammatory marker expressions, and 5-year colon cancer outcomes.

## Introduction

Analysis of differential body composition profiles has been suggested as a useful prognostic indicator in colorectal cancer (CRC).^1,2^ Baseline inflammation is increased in obesity, and the current doctrine in obesity-related cancer research is that excess visceral adiposity produces adipocytokines that fuel a proinflammatory state with an associated pro-oncogenic and prometastatic environment.^3,4,5,6^ Visceral adipocytes secrete higher levels of tumor necrosis factor α (TNFα) and interleukin (IL)-6 compared with subcutaneous adipocytes, and higher macrophage and other inflammatory cell levels have also been observed.^5,7^ These inflammatory cytokines upregulate proinflammatory pathways NF-κB, STAT3, and inhibitor of NF-κB kinase subunit β, which lead to transcription of downstream genes that are associated with mediating cancer cell proliferation, invasion, angiogenesis, cell survival, and metastatic development.^8,9,10,11^

In obesity-related cancer research, the use of body mass index (BMI; calculated as weight in kilograms divided by height in meters squared) alone to estimate oncological outcomes is conflicting, since BMI does not provide a measure of body fat distribution, particularly the difference in metabolically active visceral fat and more inert subcutaneous fat levels.^12,13^ High visceral fat is significantly proinflammatory and has been demonstrated to be an indicator associated with poor prognosis in colorectal, esophageal, gastric, kidney, bladder, and breast cancers.^5,14,15,16^ Sarcopenia is a clinical manifestation of the cancer cachexia syndrome, a catabolic state that develops owing to the metabolic demand and proinflammatory systemic state created by the presence of cancer.^17^ Sarcopenia may be potentiated by poor dietary intake in particular cancers of the gastrointestinal tract that obstruct the ability to eat or as a result of treatment adverse effects.^18,19^ Cancer cachexia syndrome requires the presence of sarcopenia, which is best measured directly on imaging (dual-energy radiographic absorptiometry or computed tomography [CT]).^20,21,22^ Therefore, sarcopenia is synonymous with the inflammatory state of cancer cachexia. Sarcopenia is consistently associated with lower survival rates in multiple cancers.^23,24,25,26^ It is also associated with reduced tolerance to and increased toxic effects from chemotherapy.^25,27^ Sarcopenia is also associated with higher postoperative complications after cancer surgery.^28,29,30^

Investigating clinically relevant associations between inflammatory pathways and body composition in a clinical setting is complex owing to the higher prevalence of underlying chronic inflammatory disorders in patients who develop cancer and the high prevalence of anti-inflammatory and immunomodulator therapy in the general population. In this study, we aimed to better understand the associations between differential body compositions, systemic inflammation, and 30-day morbidity and 5-year cancer outcomes in patients with nonmetastatic colon cancer.

## Methods

Prospective ethical approval of this study was obtained from the Clinical Research Ethics Committee of the Cork Teaching Hospitals. All patients provided written informed consent. This study is reported following the Strengthening the Reporting of Observational Studies in Epidemiology (STROBE) reporting guideline.

### Patient Recruitment, Treatment, and Follow-up

The placebo group enrolled in the Randomised Clinical Trial Assessing Use of an Anti-inflammatory Agent in Attenuating Peri-operative Inflammation in Non-metastatic Colon Cancer (S.U.R.G.U.V.A.N.T.) trial offered a unique opportunity to investigate the associations among differential body composition, inflammation, and colon cancer outcomes in a human model.^31^ Patients presenting for elective resection for colon cancer, with nonmetastatic disease, aged 18 to 85 years to 3 tertiary referral cancer centers were screened for inclusion. Extensive exclusion criteria were applied as follows: pregnant and lactating women, evidence of underlying liver disease (ie, liver function test results >2-fold reference ranges), international normalized ratio greater than 1.5, evidence of underlying kidney disease (ie, creatinine >2.36 mg/dL for women, >1.97 for men [to convert to micromoles per liter, multiply by 76.25], blood dyscrasia (neutrophils <1500 cells/μL [to convert to ×10^9^ per liter, multiply by 0.001], thrombocytes <100 × 10^3^/μL [to convert to ×10^9^ per liter, multiply by 1]), evidence of intestinal obstruction; metastases (M1: distant spread or Dukes D), morbid obesity (ie, BMI >40), operative risk greater than American Society of Anesthesiologists class III, previous cancer or malignant disease other than nonmelanoma skin cancer, coexisting active inflammatory disorder (including active rheumatoid arthritis, inflammatory bowel disease, or systemic lupus erythematosus), corticosteroids use, immunosuppressive drugs, previous diagnosis of HIV, chronic active hepatitis B or C, and active infection at the time of surgical intervention.

All patients with CRC were treated in keeping with recommendations from the Association of Coloproctologists of Great Britain and Ireland.^32^ After initial diagnosis with CRC, all patients were staged, and metastatic disease was ruled out using CT of the thorax, abdomen, and pelvis (TAP) and full endoscopy (in the absence of colonic obstruction) with tissue biopsy. All CRC operations were performed adhering to principles of oncological resection for CRC, including a no-touch technique with a high-tie of the vessels. Active surveillance continued for 5 years using a combination of clinical review, carcinoembryonic antigen testing, colonoscopy, and CT TAP, as recommended by the Association of Coloproctologists of Great Britain and Ireland.^32^

### Inflammatory Mediator Analysis

Blood samples were phlebotomized from patients preoperatively. Full blood counts were analyzed using the Sysmex XN 200 analyzer using a particle counting method based on size and density, and white cell count (WCC) was extracted from these results. Albumin levels were analyzed in our institute using a Roche Biochemistry Platform that uses a colorimetric assay (pH based). In all clinical laboratories in Ireland, National External Quality Assessment Scheme testing is performed in conjunction with daily internal control assessments to allow for accuracy of data and standardization of results. Acute phase protein and cytokine levels were measured using a standard enzyme-linked immunosorbent assay technique using a custom-made panel for the following cytokines: human IL-1b, IL-2, IL-6, IL-10, interferon (IFN) γ, TNFα, vascular endothelial growth factor (VEGF), and C-reactive protein (CRP). Neutrophil and monocyte surface receptor expression CD11b and CD14 levels were detected using flow cytometry using a FACScan flow cytometer and analyzed using the software package Cell-Quest version 5.1 (BD). The assay method involved direct staining of cell surface antigen in whole blood.

### CT Anthropometric Measurements and Categorization

CT anthropometric measurements were obtained from CT studies acquired preoperatively, usually within 4 to 6 weeks prior to surgery using a 64-slice multidetector CT (GE Healthcare). Total fat area and subcutaneous fat area were measured on a single axial CT slice 6 cm above the L4-5 intervertebral disc in centimeters-squared and visceral fat area was calculated by subtraction. Visceral fat area at this level correlates strongly with overall visceral adipose volume.^33^ Fat volume measurements were performed using the segmentation tool in the DICOM image viewer Horos (Horos Project). The full study protocol is available in eAppendix 1 in the Supplement. Visceral to total fat ratio and subcutaneous to total fat ratio were calculated. Skeletal muscle area (SMA) was measured on axial CT images at the L3 level in centimeters-squared, as this is accepted as an accurate surrogate for total skeletal muscle mass.^34,35^ Reduced SMA has been used as a surrogate for sarcopenia (an age-related or pathological state of low muscle mass and function).^36,37^ Muscle volume measurements were also performed using Horos (eAppendix 1 in the Supplement).

Visceral fat levels were categorized based on previously validated sex-specific cutoffs that have been associated with metabolic syndrome and thus are clinically significant.^38^ SMA was categorized as low at less than the 10th percentile of that observed in age- and sex-specific levels in the general population.^36^ For all other body composition profiles relating to adiposity, sex-specific median and interquartile range (IQR) values were calculated, and the parameter was categorized as the reference range at less than the 60th percentile and high at greater than the 60th percentile (50th percentile was the median). Cutoffs for each category used in this study are summarized in eAppendix 2 in the Supplement.

### Statistical Analysis

Statistical analysis was performed using SPSS version 26 (IBM), and graphs were generated using Prism version 8.4.2 (GraphPad). The association between body composition profiles and 30-day postoperative morbidity and more specifically postoperative infective complications was reported using odds ratios (ORs), 95% CIs, and *P* values generated with χ^2^ testing. Infectious complications included anastomotic leak, surgical site infection, and lower respiratory tract infection. The associations of body composition profiles with 5-year cancer recurrence and disease-specific mortality were analyzed using Mantel Cox log-rank test and Kaplan-Meier curves were produced. Hazard ratios (HRs) were generated to compare survival distributions by reference range and pathological body composition profile subgroups. Finally, when particular body composition profiles were significantly associated with poor clinical and cancer outcomes, comparison of mean inflammatory mediator expression levels was performed using Mann-Whitney *U* test, and statistical significance was set at 2-sided *P* < .05.

## Results

### Demographic and Clinicopathological Data

A total of 28 patients who underwent elective resection for colon cancer with curative intent were analyzed. Table 1 summarizes patient demographic and clinicopathological data. The median (IQR) age was 67 (58-72) years, and 22 patients (78.6%) were men. A total of 14 tumors (50.0%) were left sided, 13 tumors (46.4%) were right sided, and 1 transverse colon tumor (3.6%) was included. There were 15 T3 tumors (53.6%), 6 T4 tumors (21.4%), 5 T2 tumors (17.9%), and 2 T1 tumors (7.1%). Furthermore, 16 tumors (57.2%) were node negative, and 12 tumors (42.8%) were node positive. The median (range) nodal harvest was 15.5 (11-32) nodes. Most procedures (23 procedures [82.1%]) were completed laparoscopically, 2 procedures (7.1%) were open, and 3 procedures (10.7%) were converted from laparoscopic to open. Patients were surveyed clinically, radiologically, and endoscopically for a median (IQR) of 64 (51-95) months. Table 1 summarizes median levels of mediators of systemic inflammation and CT-derived body composition profiles with age and sex-specific categories. A total of 4 patients (14.3%) had a low SMA, and 11 patients (39.3%) had high total fat area. Differential fat measurements identified 22 patients (78.6%) with high visceral fat area and 10 patients (35.7%) with high subcutaneous fat levels. Correlation between body composition profiles and mediators of systemic inflammation and comparison of mean inflammatory level expressions based on body composition profile are summarized in the eTable in the Supplement.

**Table 1.** Summary of Patient Demographic, Clinicopathological, Inflammatory Mediator, and Body Composition Profile Data

Characteristic	No. (%) (N = 28)
Age, median (IQR) [range], y	67 (58-72) [29-80]
Sex	
Men	22 (78.6)
Women	6 (21.4)
Tumor location	
Right	13 (46.4)
Left	14 (50.0)
Transverse	1 (3.6)
Procedure	
Laparoscopic	23 (82.1)
Open	2 (7.1)
Converted	3 (10.7)
Surgery	
Anterior resection	14 (50.0)
Right hemicolectomy	13 (46.4)
Total colectomy	1 (3.6)
Primary tumor	
T1	2 (7.1)
T2	5 (17.9)
T3	15 (53.6)
T4	6 (21.4)
Nodal status	
Harvest, median (range), No.	15.5 (11-32)
Positive	12 (42.8)
Negative	16 (57.2)
Clinical outcome	
30-d morbidity	9 (32.1)
SSI	5 (17.9)
Anastomotic leak	2 (7.1)
Other	2 (7.1)
Oncological outcomes	
Recurrence	4 (14.3)
Interval, median (range), mo	22 (5-31)
Site	
Liver	3 (10.7)
Lung	1 (3.6)
Disease-specific mortality	3 (10.7)
Interval, median (range), mo	27 (12-105)
Follow-up, median (IQR), mo	64 (51-95)
Inflammatory mediators, median (IQR)	
WCC, cells/μL	7000 (5930-7770)
C-reactive protein, mg/dL	0.76 (0.25-1.04)
Albumin, g/dL	4.2 (3.8-4.5)
IFN-γ, ng/mL	2.5431 (1.9090-4.2005)
IL-1b, ng/mL	0.0758 (0.0357-0.1914)
IL-2, ng/mL	0.1815 (0.1044-0.4742)
IL-6, ng/mL	1.5803 (0.9737-2.9435)
IL-10, ng/mL	0.6377 (0.3443-4.8476)
TNFα, ng/mL	3.8541 (2.9892-5.2239)
VEGF, ng/mL	310.695 (197.602-690.948)
CD14, ng/mL	387.370 (22.893-690.948)
CD11b, ng/mL	155.455 (23.94-485.795)
Skeletal muscle area	
Median (IQR), cm^2^	150.2 (135.03-163.88)
Within reference range	24 (85.7)
Low	4 (14.3)
Total fat	
Median (IQR), cm^2^	379.1 (297.35-531.33)
High	11 (39.3)
Within reference range	17 (60.7)
Subcutaneous fat	
Median (IQR), cm^2^	185.8 (145.30-230.45)
High	10 (35.7)
Within reference range	18 (64.3)
Visceral fat	
Median (IQR), cm^2^	183.5 (121.10-302.13)
High	22 (78.6)
Within reference range	6 (21.4)
Visceral to total fat ratio	
Median (IQR)	0.533 (0.396-0.587)
High	21 (75.0)
Within reference range	7 (25.0)
Subcutaneous to total fat ratio	
Median (IQR)	0.469 (0.413-0.604)
High	11 (39.3)
Within reference range	17 (60.7)

Abbreviations: IFN, interferon; IL, interleukin; IQR, interquartile range; SSI, surgical site infection; TNFα, tumor necrotic factor α; VEGF, vascular endothelial growth factor; WCC, white cell count.

SI conversion factors: To convert albumin to grams per liter, multiply by 10; C-reactive protein to milligrams per liter, multiply by 10; WCC to ×10^9^ per liter, multiply by 0.001.

### Clinical and Oncological Outcomes

Nine patients (32.1%) developed a postoperative complication within the first 30 days postoperatively (Table 1), including 5 patients (17.9%) who developed a surgical site infection, 2 patients (7.1%) who developed an anastomotic leak, both requiring a return to surgery, 1 patient (3.6%) who developed a lower respiratory tract infection, and 1 patient (3.6%) who developed prolonged postoperative ileus (ie, persistent ileus >48 hours postoperatively). A total of 4 patients experienced cancer recurrence at a median (range) interval of 22 (5-31) months. All were distant recurrence, including 3 hepatic tumors and 1 pulmonary tumor. A total of 3 patients (10.7%) experienced disease-specific mortality. The median (range) interval from surgery to mortality was 27 (51-95) months.

### Associations of Body Composition Profiles With Colon Cancer Outcomes

**Figure 1.**
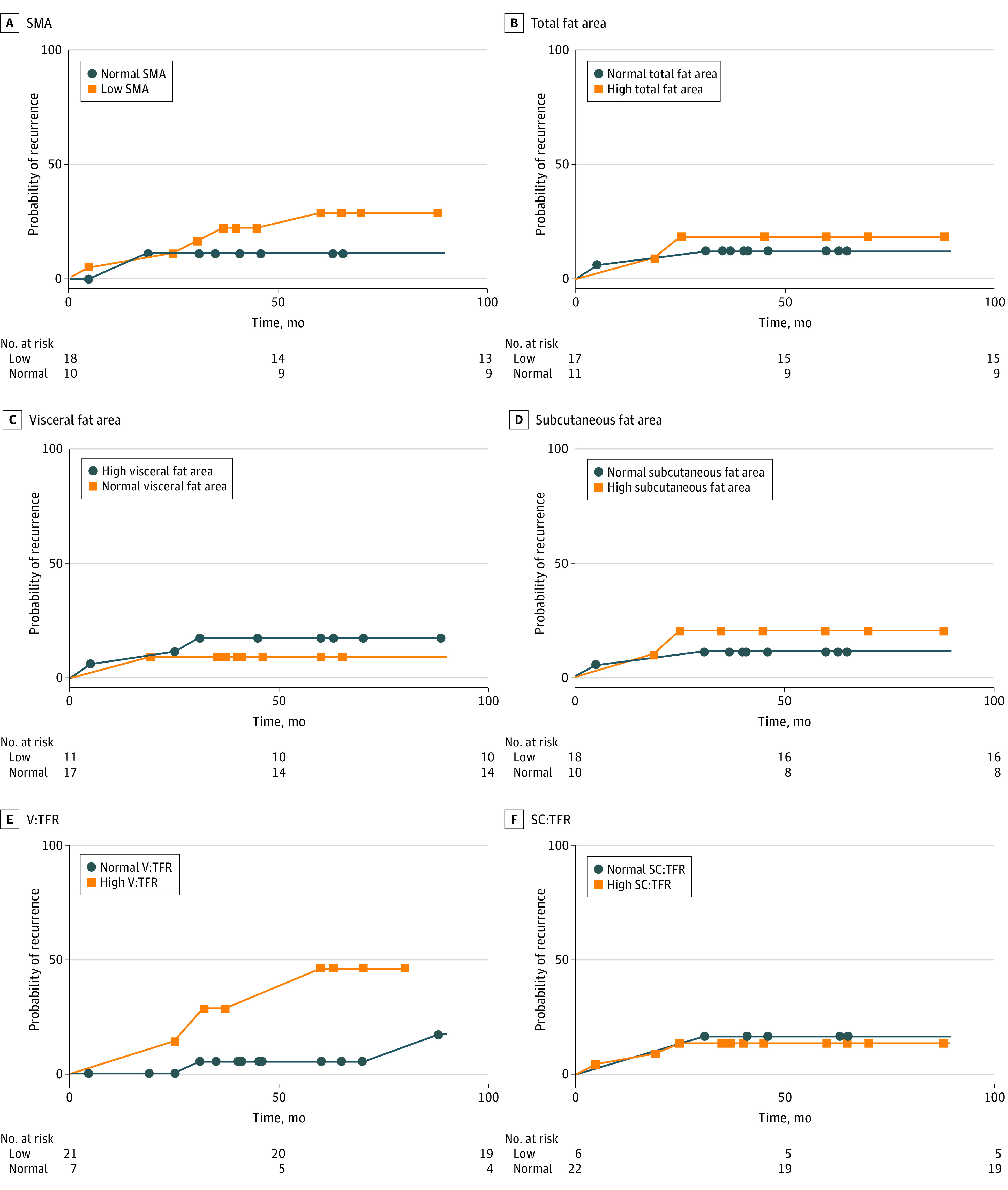
Risk of 5-Year Colon Cancer Recurrence Based on Body Composition Profiles SC:TFR indicates subcutaneous to total fat ratio; SMA, skeletal muscle area; and V:TFR, visceral to total fat ratio. Box and circle markers indicate a recurrence when associated with a change in direction of the linear line or a point of censorship (ie, the time at which follow-up ceased).

**Figure 2.**
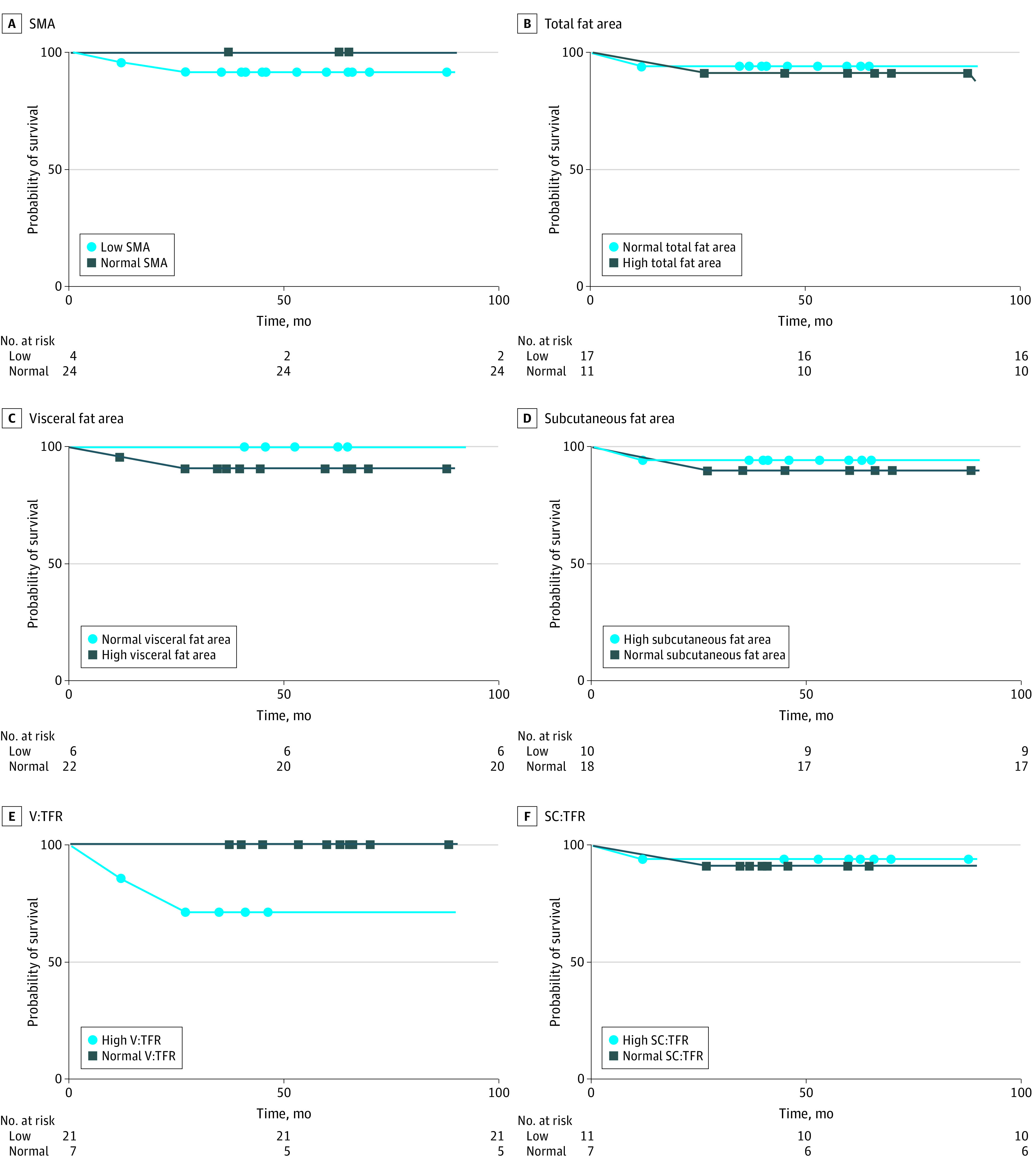
Risk of 5-Year Disease-Specific Mortality in Colon Cancer Based on Body Composition Profiles SC:TFR indicates subcutaneous to total fat ratio; SMA, skeletal muscle area; and V:TFR, visceral to total fat ratio. Box and circle markers indicate a disease-specific mortality or a point of censorship (ie, the time at which follow-up ceased).

Low SMA and high visceral to total fat ratio were associated with less favorable clinical and oncological outcomes. There was no significant association between differential body composition profiles and overall 30-day morbidity. Low SMA (OR, 2.13 [95% CI, 1.85-5.36]; *P* = .004) and high visceral to total fat ratio (OR, 3.20 [95% CI, 1.85-10.84]; *P* = .01] were significantly associated with developing a 30-day infective complication (Table 2). A log-rank Mantel test was performed to analyze the association of differential body composition profiles with 5-year colon cancer recurrence (Figure 1). Low SMA (HR, 2.30 [95% CI, 1.41-2.89]; *P* = .04) and high visceral to total fat ratio (HR, 5.78 [95% CI, 3.66-7.95]; *P* = .02) were significantly associated with developing a cancer recurrence within the first 5 years after surgery. A log-rank Mantel test was performed to analyze the association of differential body composition profiles with 5-year disease-specific mortality (Figure 2). High visceral to total fat ratio (HR, 5.92 [95% CI, 4.04-8.00]; *P* = .02) was the only body composition profile significantly associated with cancer-related mortality within the first 5 years after surgery. There was no significant association between low SMA and 5-year disease-specific mortality (HR, 3.22 [95% CI, 0.13-8.37]; *P* = .48).

**Table 2.** Associations of Body Composition Profiles With 30-Day Morbidity and Postoperative Infectious Complications After Surgery for Nonmetastatic Colon Cancer

Body composition	30-d morbidity		Postoperative infective complications	
	OR (95% CI)^a^	*P* value	OR (95% CI)^a^	*P* value
Low SMA	1.52 (0.24-9.76)	.55	2.13 (1.85-5.36)	.04
Total fat area	1.40 (0.77-2.56)	.21	1.51 (0.84-2.70)	.12
Visceral fat area	1.22 (0.77-1.93)	.43	1.30 (0.81-2.11)	.35
Subcutaneous fat area	1.39 (0.88-2.18)	.25	1.67 (0.41-6.77)	.39
Visceral to total fat ratio	1.94 (0.50-7.64)	.28	3.20 (1.95-10.84)	.01
Subcutaneous to total fat ratio	1.29 (0.41-4.13)	.49	1.08 (0.32-3.63)	.62

Abbreviations: OR, odds ratio; SMA, skeletal muscle area.

^a^ ORs are calculated as high vs low. Visceral fat levels were categorized based on previously validated sex-specific cutoffs that have been associated with metabolic syndrome and thus are clinically significant. SMA was categorized as low at less than the 10th percentile of that observed in age- and sex-specific levels in the general population. For all other body composition profiles relating to adiposity, sex-specific median and interquartile range values were calculated, and the parameter was categorized as the reference range at less than the 60th percentile and high at greater than the 60th percentile (50th percentile was the median). Cutoffs for each category are summarized in eAppendix 2 in the Supplement.

### Systemic Inflammation, Body Composition Profiles, and Outcome Analysis

**Figure 3.**
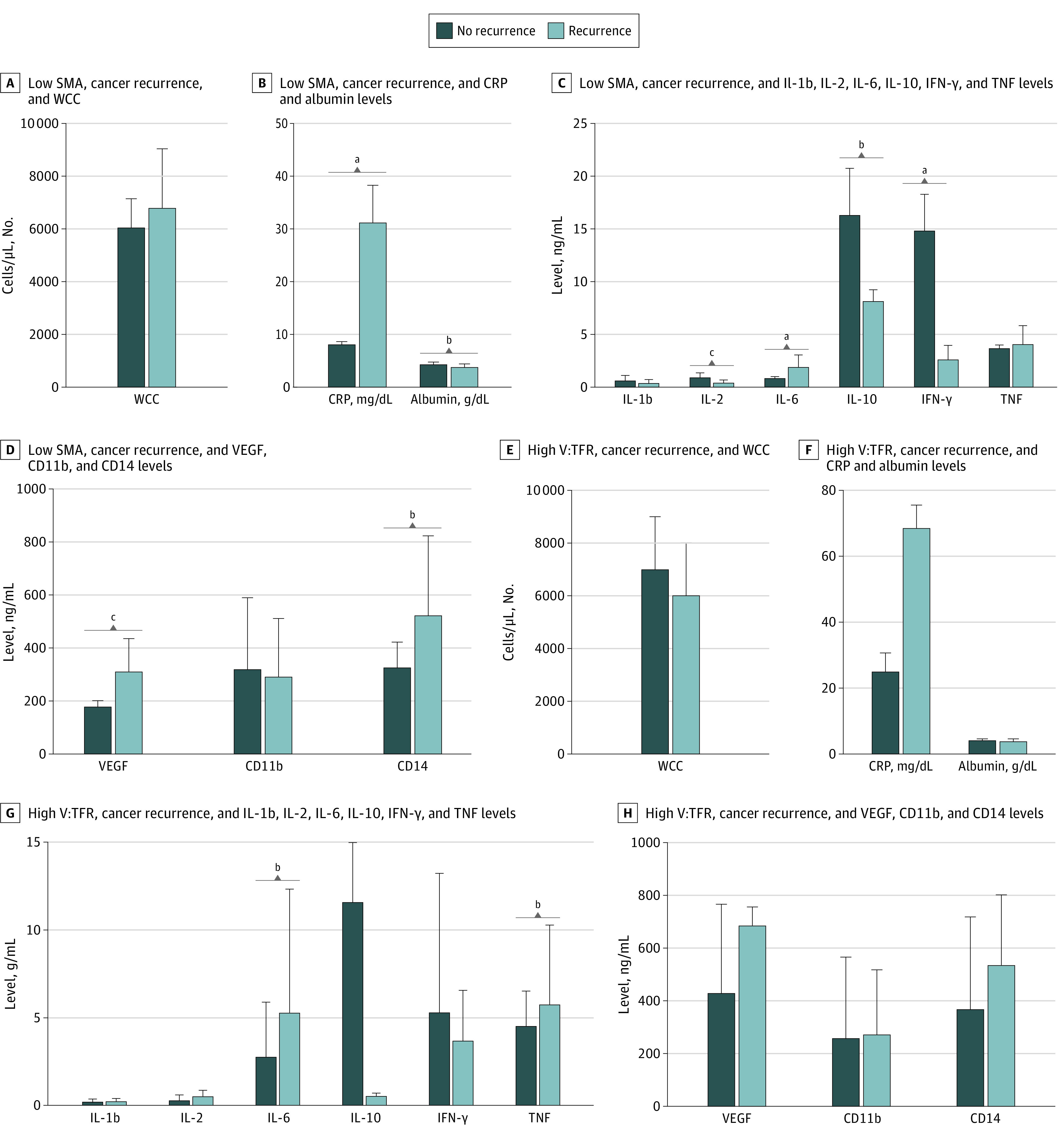
Comparison of Mean Inflammatory Mediator Levels in Patients Who Developed Cancer Recurrence and Those Who Did Not CRP indicates C-reactive protein; IFN, interferon; IL, interleukin; SMA, skeletal muscle area; TNF, tumor necrosis factor; V:TFR, visceral to total fat ratio; VEGF, vascular endothelial growth factor; and WCC, white cell count. SI conversion factors: To convert albumin to grams per liter, multiply by 10; CRP to milligrams per liter, multiply by 10; WCC to ×10^9^ per liter, multiply by 0.001. ^a^*P* < .01. ^b^*P* < .05. ^c^*P* < .001.

Patients with low SMA who developed an infectious complication, compared with those who did not, had significantly higher WCC (mean [SD], 7100 [1870] cells/μL vs 5600 [1580] cells/μL [to convert to ×10^9^/L, multiply by 0.001]; *P* = .04) and CRP (mean [SD], 3.29 [2.52] mg/dL vs 0.70 [0.10] mg/dL [to convert to milligrams per liter, multiply by 10]; *P* = .01) levels, higher expression of IL-10 (mean [SD], 8.75 [5.79] ng/mL vs 0.42 [0.24] ng/mL; *P* = .004), VEGF (mean [SD], 298.42 [134.90] ng/mL vs 183.86 [23.67] ng/mL; *P* = .004), and CD14 (mean [SD], 521.23 [302.02] ng/mL vs 322.07 [98.35] ng/mL; *P* = .03), and lower levels of IFN-γ (mean [SD], 1.38 [1.62] ng/mL vs 3.22 [1.15] ng/mL; *P* = .046) (eFigure 1 in the Supplement). Patients with high visceral to total fat ratio who developed infectious complications, compared with those who did not, had significantly higher CRP (mean [SD], 5.30 [0.88] mg/dL vs 1.09 [1.40] mg/dL; *P* = .001) and IFN-γ (mean [SD], 7.76 [1.41] ng/mL vs 2.89 [1.84] ng/mL; *P* = .01) and lower expression of IL-2 (mean [SD], 0.12 [0.09] ng/mL vs 0.41 [0.31] ng/mL; *P* = .03). Figure 3 shows that patients with low SMA who developed cancer recurrence, compared with those who did not, had significantly higher levels of CRP (mean [SD], 31.24 [6.95] mg/dL vs 8.11 [0.58] mg/dL; *P* = .003), IL-6 (mean [SD], 1.93 [1.16] ng/mL vs 0.88 [0.14] ng/mL; *P* = .004), VEGF (mean [SD], 310.03 [122.66] ng/mL vs 176.12 [22.94] ng/mL; *P* = .007), and CD14 (mean [SD], 521.23 [302.02] ng/mL vs 322.07 [98.35] ng/mL; *P* = .03) expression. Significantly lower levels of albumin (mean [SD], 3.8 [0.6] g/dL vs 4.4 [3.7] g/dL [to convert to grams per liter, multiply by 10]; *P* = .01), IL-2 (mean [SD], 0.45 [0.25] ng/mL vs 0.94 [0.43] ng/mL; *P* < .001), IL-10 (mean [SD], 8.15 [1.09] ng/mL vs 16.32 [4.43] ng/mL; *P* = .004), and IFN-γ (mean [SD], 2.61 [1.36] ng/mL vs 14.87 [3.43] ng/mL; *P* = .02) were also observed. Patients with high visceral to total fat ratio who developed recurrence, compared with those who did not, had higher levels of IL-6 (mean [SD], 5.26 [7.05] ng/mL vs 2.76 [3.11] ng/mL; *P* = .03) and TNFα (mean [SD], 5.74 [4.53] ng/mL vs 4.50 [1.99] ng/mL; *P* = .03). There was no significant difference in systemic inflammatory mediator expression levels in patients who had high visceral to total fat ratio among those who experienced disease-specific mortality vs those who were disease-free at 5 years (eFigure 2 in the Supplement).

## Discussion

This cohort study found that low SMA and high visceral to total fat ratio were associated with increased postoperative infections and higher 5-year cancer recurrence and disease-specific mortality rates in patients with CRC. High visceral to total fat ratio was significantly associated with poor outcomes compared with high total body fat or visceral fat levels alone. Interestingly, subcutaneous fat levels correlated with IL-2 and IL-10 levels, ie, cytokines proposed to possess predominantly anti-inflammatory as opposed to proinflammatory properties. Thus higher subcutaneous fat area may have a protective association with the deleterious inflammatory outcomes associated with visceral fat. This phenomenon may be why BMI is not a reliable prognostic marker in cancer and cancer surgery, as it does not reflect differential fat distribution or muscle mass distribution.^20^

In obesity-related cancer research, BMI alone is not associated with predicting oncological outcomes, as BMI does not reflect body fat distribution, particularly the difference between metabolically active visceral fat and more inert subcutaneous fat levels.^12,13^ This is sometimes referred to as the *obesity paradox in cancer*, in which high sex-specific BMI levels are not always associated with poor prognosis in cancer.^15^ In CRC, increased visceral adiposity is associated with worse operative outcomes, including increased postoperative complications after resection,^1,39,40^ surgical site infection,^1,39,41^ and anastomotic leakage rates.^1^ In rectal cancer, high visceral fat levels are associated with lower lymph node harvests,^42^ less favorable tumor response to neoadjuvant therapy,^43,44^ and a reduction in disease-free and overall survival rates across all stages of CRC.^41,42,44,45,46,47,48^ In this study, we found that subcutaneous fat was associated with higher levels of cytokines with protective associations, including IL-2 and IL-10. This may explain why high visceral to total fat ratio was more prognostic of poor clinical outcomes (ie, postoperative infectious complications) and oncological outcomes (ie, 5-year cancer recurrence and disease-specific mortality) than visceral fat levels alone, as higher levels of protective cytokines abundant in subcutaneous fat may counteract the inflammasomes of visceral fat and neutralize their systemic effect.

Sarcopenia is described as a clinical manifestation of the cancer cachexia syndrome.^17^ CT analysis is increasingly used to assess surrogates of sarcopenia, including SMA, skeletal muscle index, and muscle radiation attenuation.^36^ In this cohort study, we found that low SMA was significantly associated with postoperative infectious complications and 5-year cancer recurrence. In patients who developed cancer recurrence and had low SMA on staging CT TAP, significantly higher systemic levels of CRP, IL-6, VEGF, and expression of cell surface receptor CD14 were observed. These inflammatory mediators and cell receptors are known to promote cancer cell survival and metastatic transformation. Interestingly, in these patients, there were also significantly lower levels of IL-2 and IL-10.

Expression levels of CD11b and CD14 displayed an inverse association with total fat area, visceral fat area, and visceral to total fat ratio and a direct association with SMA, subcutaneous fat area, and subcutaneous to total fat ratio in all patients, and significantly higher expression of CD14 was observed in patients with low SMA who developed postoperative infections and 5-year cancer recurrence. Both receptors are expressed mainly by mononuclear phagocytes (monocytes and macrophages) but also neutrophils and dendritic cells.^49,50^ Mononuclear phagocytes represent the principal target for lipopolysaccharides of gram-negative bacteria. Lipopolysaccharides trigger a wide range of cellular responses, including the synthesis and release of a variety of inflammatory mediators, such as TNFα, IL-1b, and IL-6, which, if unregulated, can promote oncogenesis and metastatic development.^49^ The triggering of cellular responses is initiated by binding lipopolysaccharides to the cell surface via cell surface receptors (eg, CD11b and CD14) and its subsequent internalisation.^50^ Higher expression of CD14 in the setting of low SMA promotes lipopolysaccharide binding and development of infection that may contribute to activation of dormant circulating tumor cells and cellular migration and invasion for metastatic development.

The question remains on whether targeted therapies can modulate the inflammatory association of body composition with cancer outcomes. Recently, the concept of prehabiliation has gained significant interest in CRC surgery, and, considering that the body composition profiles and inflammatory markers reported in this study were performed preoperatively, it raises the question as to whether prehabilitation could reverse this association. Prehabilitation involves physical, nutritional, and psychosocial optimization preoperatively, with an aim to reduce operative morbidity and promote recovery.^51^ However, outcomes from prehabilitation programs have been conflicting to date, mostly owing to difficulty in identifying the optimum interventions and measures of success.^51^ To our knowledge, no specific nutritional intervention has been identified as effective in reversing the effects of sarcopenia.^52^ The reason for this has been suggested as the catabolic effect of the primary tumor remaining in situ and the inability of nutritional efforts to overcome this.^17^ Some targeted anti-inflammatory therapies, including anti–IL-6 therapy or combined β-blockade and cyclooxygenase 2 inhibition, have shown promising results at counteracting outcomes associated with perioperative inflammation; however, whether this can be extrapolated to modulate the effect of body composition preoperatively has not been reported, to our knowledge.^31,53,54,55^

### Limitations

There are several limitations to this study. This was a retrospective analysis of the placebo group in a prospective randomized clinical trial, with the associated limitations of such a study design. It also included a small sample size of 28 patients, and we propose the findings of this work as exploratory and preliminary. Furthermore, it is important to recognize that the study cohort included more men (78.6%) than women (21.4%). However, although small in number, the extensive exclusion criteria supported a homogenous group of patients with CRC in whom to purely study the associations of inflammation. The incidences of 30-day morbidity and cancer outcomes were also small owing to the limited sample size. Infectious complications were grouped for outcome analysis to overcome some of this limitation, since it is known that any infectious complication can affect cancer outcomes.^56,57^

## Conclusions

This cohort study found that low SMA and high visceral to total fat ratio were associated with increased risk of postoperative infections and 5-year cancer recurrence; high visceral to total fat ratio was further associated with 5-year disease-specific mortality. Furthermore, upregulation of proinflammatory cytokines, acute phase proteins, and VEGF and downregulation of protective anti-inflammatory mediators was pronounced in patients with low SMA and high visceral to total fat ratio who experienced poor clinical or cancer outcomes. Therefore, these findings suggest that the negative impact of sarcopenia and visceral fat on cancer outcomes may be driven by systemic inflammation.

## Notes

Supplement. **eAppendix 1.** Full Method Used to Calculate Body Composition Profiles on Axial Computed Tomography **eAppendix 2.** Sex-Specific Cutoff Values for Reference Range and Pathological Body Composition Parameter Subgroups **eTable.** Summary of Comparison of Means and Correlation of Individual Body Composition Profiles With Systemic Mediators of Inflammation **eFigure 1.** Comparison of Mean Inflammatory Mediator Levels in Patients Who Developed a Postoperative Complication and Those Who Did Not **eFigure 2.** Comparison of Mean Inflammatory Mediator Levels in Patients Who Experienced Disease-Specific Mortality and Those Who Did Not in Patients With High Visceral to Total Fat Ratio (V:TFR)

## References

[1] Watanabe J, Tatsumi K, Ota M, . The impact of visceral obesity on surgical outcomes of laparoscopic surgery for colon cancer. Int J Colorectal Dis. 2014;29(3):343-351. doi:10.1007/s00384-013-1803-9 24297037

[2] Black D, Mackay C, Ramsay G, . Prognostic value of computed tomography: measured parameters of body composition in primary operable gastrointestinal cancers. Ann Surg Oncol. 2017;24(8):2241-2251. doi:10.1245/s10434-017-5829-z 28324283 PMC5491683

[3] Balkwill F, Mantovani A. Inflammation and cancer: back to Virchow? Lancet. 2001;357(9255):539-545. doi:10.1016/S0140-6736(00)04046-0 11229684

[4] Mantovani A, Garlanda C, Allavena P. Molecular pathways and targets in cancer-related inflammation. Ann Med. 2010;42(3):161-170. doi:10.3109/07853890903405753 20384432

[5] Donohoe CL, Doyle SL, Reynolds JV. Visceral adiposity, insulin resistance and cancer risk. Diabetol Metab Syndr. 2011;3(1):12. doi:10.1186/1758-5996-3-12 21696633 PMC3145556

[6] Martinez-Useros J, Garcia-Foncillas J. Obesity and colorectal cancer: molecular features of adipose tissue. J Transl Med. 2016;14(1):21. doi:10.1186/s12967-016-0772-5 26801617 PMC4722674

[7] Doyle SL, Donohoe CL, Lysaght J, Reynolds JV. Visceral obesity, metabolic syndrome, insulin resistance and cancer. Proc Nutr Soc. 2012;71(1):181-189. doi:10.1017/S002966511100320X 22051112

[8] Sonnenberg GE, Krakower GRKA, Kissebah AH. A novel pathway to the manifestations of metabolic syndrome. Obes Res. 2004;12(2):180-186. doi:10.1038/oby.2004.24 14981209

[9] Aggarwal BB, Vijayalekshmi RV, Sung B. Targeting inflammatory pathways for prevention and therapy of cancer: short-term friend, long-term foe. Clin Cancer Res. 2009;15(2):425-430. doi:10.1158/1078-0432.CCR-08-0149 19147746

[10] O’Leary DP, Wang JH, Cotter TG, Redmond HP. Less stress, more success: oncological implications of surgery-induced oxidative stress. Gut. 2013;62(3):461-470. doi:10.1136/gutjnl-2011-300948 22147551

[11] O’Leary DP, O’Leary E, Foley N, Cotter TG, Wang JH, Redmond HP. Effects of surgery on the cancer stem cell niche. Eur J Surg Oncol. 2016;42(3):319-325. doi:10.1016/j.ejso.2015.12.008 26810247

[12] Dignam JJ, Polite BN, Yothers G, . Body mass index and outcomes in patients who receive adjuvant chemotherapy for colon cancer. J Natl Cancer Inst. 2006;98(22):1647-1654. doi:10.1093/jnci/djj442 17105987

[13] Meyerhardt JA, Niedzwiecki D, Hollis D, ; Cancer and Leukemia Group B 89803. Impact of body mass index and weight change after treatment on cancer recurrence and survival in patients with stage III colon cancer: findings from Cancer and Leukemia Group B 89803. J Clin Oncol. 2008;26(25):4109-4115. doi:10.1200/JCO.2007.15.6687 18757324 PMC2654367

[14] Malietzis G, Aziz O, Bagnall NM, Johns N, Fearon KC, Jenkins JT. The role of body composition evaluation by computerized tomography in determining colorectal cancer treatment outcomes: a systematic review. Eur J Surg Oncol. 2015;41(2):186-196. doi:10.1016/j.ejso.2014.10.056 25468746

[15] Lennon H, Sperrin M, Badrick E, Renehan AG. The obesity paradox in cancer: a review. Curr Oncol Rep. 2016;18(9):56. doi:10.1007/s11912-016-0539-4 27475805 PMC4967417

[16] Moore SC, Playdon MC, Sampson JN, . A metabolomics analysis of body mass index and postmenopausal breast cancer risk. J Natl Cancer Inst. 2018;110(6):588-597. doi:10.1093/jnci/djx244 29325144 PMC6279273

[17] Ryan AM, Power DG, Daly L, Cushen SJ, Ní Bhuachalla Ē, Prado CM. Cancer-associated malnutrition, cachexia and sarcopenia: the skeleton in the hospital closet 40 years later. Proc Nutr Soc. 2016;75(2):199-211. doi:10.1017/S002966511500419X 26786393

[18] Blum D, Omlin A, Baracos VE, ; European Palliative Care Research Collaborative. Cancer cachexia: a systematic literature review of items and domains associated with involuntary weight loss in cancer. Crit Rev Oncol Hematol. 2011;80(1):114-144. doi:10.1016/j.critrevonc.2010.10.004 21216616

[19] Bye A, Jordhøy MS, Skjegstad G, Ledsaak O, Iversen PO, Hjermstad MJ. Symptoms in advanced pancreatic cancer are of importance for energy intake. Support Care Cancer. 2013;21(1):219-227. doi:10.1007/s00520-012-1514-8 22684989

[20] Fearon K, Strasser F, Anker SD, . Definition and classification of cancer cachexia: an international consensus. Lancet Oncol. 2011;12(5):489-495. doi:10.1016/S1470-2045(10)70218-7 21296615

[21] Mourtzakis M, Prado CMLJ, Lieffers JR, Reiman T, McCargar LJ, Baracos VE. A practical and precise approach to quantification of body composition in cancer patients using computed tomography images acquired during routine care. Appl Physiol Nutr Metab. 2008;33(5):997-1006. doi:10.1139/H08-075 18923576

[22] Shen W, Punyanitya M, Wang Z, . Total body skeletal muscle and adipose tissue volumes: estimation from a single abdominal cross-sectional image. J Appl Physiol (1985). 2004;97(6):2333-2338. doi:10.1152/japplphysiol.00744.2004 15310748

[23] Choi MH, Oh SN, Lee IK, Oh ST, Won DD. Sarcopenia is negatively associated with long-term outcomes in locally advanced rectal cancer. J Cachexia Sarcopenia Muscle. 2018;9(1):53-59. doi:10.1002/jcsm.12234 28849630 PMC5803619

[24] Peng P, Hyder O, Firoozmand A, . Impact of sarcopenia on outcomes following resection of pancreatic adenocarcinoma. J Gastrointest Surg. 2012;16(8):1478-1486. doi:10.1007/s11605-012-1923-5 22692586 PMC3578313

[25] Antoun S, Baracos VE, Birdsell L, Escudier B, Sawyer MB. Low body mass index and sarcopenia associated with dose-limiting toxicity of sorafenib in patients with renal cell carcinoma. Ann Oncol. 2010;21(8):1594-1598. doi:10.1093/annonc/mdp605 20089558

[26] Sharma P, Zargar-Shoshtari K, Caracciolo JT, . Sarcopenia as a predictor of overall survival after cytoreductive nephrectomy for metastatic renal cell carcinoma. Urol Oncol. 2015;33(8):339.e17-339.e23. doi:10.1016/j.urolonc.2015.01.011 26094169

[27] Cushen SJ, Power DG, Murphy KP, . Impact of body composition parameters on clinical outcomes in patients with metastatic castrate-resistant prostate cancer treated with docetaxel. Clin Nutr ESPEN. 2016;13:e39-e45. doi:10.1016/j.clnesp.2016.04.001 28531567

[28] Elliott JA, Doyle SL, Murphy CF, . Sarcopenia: prevalence, and impact on operative and oncologic outcomes in the multimodal management of locally advanced esophageal cancer. Ann Surg. 2017;266(5):822-830. doi:10.1097/SLA.0000000000002398 28796017

[29] Wagner D, DeMarco MM, Amini N, . Role of frailty and sarcopenia in predicting outcomes among patients undergoing gastrointestinal surgery. World J Gastrointest Surg. 2016;8(1):27-40. doi:10.4240/wjgs.v8.i1.27 26843911 PMC4724585

[30] O’Brien S, Twomey M, Moloney F, . Sarcopenia and post-operative morbidity and mortality in patients with gastric cancer. J Gastric Cancer. 2018;18(3):242-252. doi:10.5230/jgc.2018.18.e25 30276001 PMC6160525

[31] Redmond HP, Neary PM, Jinih M, . Randomised Clinical Trial Assessing Use of an Anti-inflammatory Agent in Attenuating Peri-operative Inflammation in Non-metastatic Colon Cancer—the S.U.R.G.U.V.A.N.T. trial. BMC Cancer. 2018;18(1):794. doi:10.1186/s12885-018-4641-x 30081854 PMC6091184

[32] Leong K, Hartley J, Karandikar S. Association of coloproctology of Great Britain & Ireland (ACPGBI): guidelines for the management of cancer of the colon, rectum and anus (2017)—follow up, lifestyle and survivorship. Colorectal Dis. 2017;19(suppl 1):67-70. doi:10.1111/codi.13706 28632315

[33] Demerath EW, Shen W, Lee M, . Approximation of total visceral adipose tissue with a single magnetic resonance image. Am J Clin Nutr. 2007;85(2):362-368. doi:10.1093/ajcn/85.2.362 17284730 PMC2883309

[34] Dello SA, Lodewick TM, van Dam RM, . Sarcopenia negatively affects preoperative total functional liver volume in patients undergoing liver resection. HPB (Oxford). 2013;15(3):165-169. doi:10.1111/j.1477-2574.2012.00517.x 23020663 PMC3572275

[35] Lieffers JR, Bathe OF, Fassbender K, Winget M, Baracos VE. Sarcopenia is associated with postoperative infection and delayed recovery from colorectal cancer resection surgery. Br J Cancer. 2012;107(6):931-936. doi:10.1038/bjc.2012.350 22871883 PMC3464761

[36] van der Werf A, Langius JAE, de van der Schueren MAE, . Percentiles for skeletal muscle index, area and radiation attenuation based on computed tomography imaging in a healthy Caucasian population. Eur J Clin Nutr. 2018;72(2):288-296. doi:10.1038/s41430-017-0034-5 29242526 PMC5842880

[37] Cruz-Jentoft AJ, Baeyens JP, Bauer JM, ; European Working Group on Sarcopenia in Older People. Sarcopenia: European consensus on definition and diagnosis: report of the European Working Group on Sarcopenia in Older People. Age Ageing. 2010;39(4):412-423. doi:10.1093/ageing/afq034 20392703 PMC2886201

[38] Doyle SL, Bennett AM, Donohoe CL, . Establishing computed tomography-defined visceral fat area thresholds for use in obesity-related cancer research. Nutr Res. 2013;33(3):171-179. doi:10.1016/j.nutres.2012.12.007 23507222

[39] Tsujinaka S, Konishi F, Kawamura YJ, . Visceral obesity predicts surgical outcomes after laparoscopic colectomy for sigmoid colon cancer. Dis Colon Rectum. 2008;51(12):1757-1765. doi:10.1007/s10350-008-9395-0 18600376

[40] Ishii Y, Hasegawa H, Nishibori H, Watanabe M, Kitajima M. Impact of visceral obesity on surgical outcome after laparoscopic surgery for rectal cancer. Br J Surg. 2005;92(10):1261-1262. doi:10.1002/bjs.5069 16078294

[41] Rickles AS, Iannuzzi JC, Mironov O, . Visceral obesity and colorectal cancer: are we missing the boat with BMI? J Gastrointest Surg. 2013;17(1):133-143. doi:10.1007/s11605-012-2045-9 23090279

[42] Ballian N, Lubner MG, Munoz A, . Visceral obesity is associated with outcomes of total mesorectal excision for rectal adenocarcinoma. J Surg Oncol. 2012;105(4):365-370. doi:10.1002/jso.22031 21751219

[43] Guiu S, Mouret Reynier MA, Toure M, Coudert B. Predictive factors of response in HER2-positive breast cancer treated by neoadjuvant therapy. J Oncol. 2013;2013:854121. doi:10.1155/2013/854121 23737784 PMC3657410

[44] Clark W, Siegel EM, Chen YA, . Quantitative measures of visceral adiposity and body mass index in predicting rectal cancer outcomes after neoadjuvant chemoradiation. J Am Coll Surg. 2013;216(6):1070-1081. doi:10.1016/j.jamcollsurg.2013.01.007 23523147 PMC4621808

[45] Xiao J, Mazurak VC, Olobatuyi TA, Caan BJ, Prado CM. Visceral adiposity and cancer survival: a review of imaging studies. Eur J Cancer Care (Engl). 2018;27(2):e12611. doi:10.1111/ecc.12611 27921375

[46] Lee CS, Murphy DJ, McMahon C, . Visceral adiposity is a risk factor for poor prognosis in colorectal cancer patients receiving adjuvant chemotherapy. J Gastrointest Cancer. 2015;46(3):243-250. doi:10.1007/s12029-015-9709-0 25832480

[47] Guiu B, Petit JM, Bonnetain F, . Visceral fat area is an independent predictive biomarker of outcome after first-line bevacizumab-based treatment in metastatic colorectal cancer. Gut. 2010;59(3):341-347. doi:10.1136/gut.2009.188946 19837679

[48] Moon H-G, Ju Y-T, Jeong C-Y, . Visceral obesity may affect oncologic outcome in patients with colorectal cancer. Ann Surg Oncol. 2008;15(7):1918-1922. doi:10.1245/s10434-008-9891-4 18392660

[49] Cheah MT, Chen JY, Sahoo D, . CD14-expressing cancer cells establish the inflammatory and proliferative tumor microenvironment in bladder cancer. Proc Natl Acad Sci U S A. 2015;112(15):4725-4730. doi:10.1073/pnas.1424795112 25825750 PMC4403197

[50] Hmama Z, Mey A, Normier G, Binz H, Revillard JP. CD14 and CD11b mediate serum-independent binding to human monocytes of an acylpolygalactoside isolated from Klebsiella pneumoniae. Infect Immun. 1994;62(5):1520-1527. doi:10.1128/IAI.62.5.1520-1527.1994 7513300 PMC186345

[51] Thomas G, Tahir MR, Bongers BC, Kallen VL, Slooter GD, van Meeteren NL. Prehabilitation before major intra-abdominal cancer surgery: a systematic review of randomised controlled trials. Eur J Anaesthesiol. 2019;36(12):933-945. doi:10.1097/EJA.0000000000001030 31188152 PMC6855314

[52] Baldwin C, Spiro A, Ahern R, Emery PW. Oral nutritional interventions in malnourished patients with cancer: a systematic review and meta-analysis. J Natl Cancer Inst. 2012;104(5):371-385. doi:10.1093/jnci/djr556 22345712

[53] Shaashua L, Shabat-Simon M, Haldar R, . Perioperative COX-2 and β-adrenergic blockade improves metastatic biomarkers in breast cancer patients in a phase-II randomized trial. Clin Cancer Res. 2017;23(16):4651-4661. doi:10.1158/1078-0432.CCR-17-0152 28490464 PMC5559335

[54] Sooriakumaran P, Coley HM, Fox SB, . A randomized controlled trial investigating the effects of celecoxib in patients with localized prostate cancer. Anticancer Res. 2009;29(5):1483-1488. 19443354

[55] Dhawan D, Craig BA, Cheng L, . Effects of short-term celecoxib treatment in patients with invasive transitional cell carcinoma of the urinary bladder. Mol Cancer Ther. 2010;9(5):1371-1377. doi:10.1158/1535-7163.MCT-10-0049 20423998 PMC2868069

[56] Clancy C, O’Leary DP, Burke JP, . A meta-analysis to determine the oncological implications of conversion in laparoscopic colorectal cancer surgery. Colorectal Dis. 2015;17(6):482-490. doi:10.1111/codi.12875 25524157

[57] Beecher SM, OʼLeary DP, McLaughlin R, Kerin MJ. The impact of surgical complications on cancer recurrence rates: a literature review. Oncol Res Treat. 2018;41(7-8):478-482. doi:10.1159/000487510 29895008

