# Supplementary material for: Body Composition, Inflammation, and 5-Year Outcomes in Colon Cancer
Source: JAMA Netw Open. 2021 Aug 30;4(8):e2115274. doi: 10.1001/jamanetworkopen.2021.15274 (PMC8406082; doi:10.1001/jamanetworkopen.2021.15274)
Supplement: Supplement. — eAppendix 1. Full Method Used to Calculate Body Composition Profiles on Axial Computed Tomography eAppendix 2. Sex-Specific Cutoff Values for Reference Range and Pathological Body Composition Parameter Subgroups eTable. Summary of Comparison of Means and Correlation of Individual Body Composition Profiles With Systemic Mediators of Inflammation eFigure 1. Comparison of Mean Inflammatory Mediator Levels in Patients Who Developed a Postoperative Complication and Those Who Did Not eFigure 2. Comparison of Mean Inflammatory Mediator Levels in Patients Who Experienced Disease-Specific Mortality and Those Who Did Not in Patients With High Visceral to Total Fat Ratio (V:TFR) [file jamanetwopen-e2115274-s001.pdf]

Supplemental Online Content

Fleming CA, O'Connell EP, Kavanagh RG, et al. Body composition, inflammation, and 5-year outcomes in colon cancer. *JAMA Netw Open*. 2021;4(7):e2115274. doi:10.1001/jamanetworkopen.2021.15274

eAppendix 1. Full Method Used to Calculate Body Composition Profiles on Axial Computed Tomography
eAppendix 2. Sex-Specific Cutoff Values for Reference Range and Pathological Body Composition Parameter Subgroups
eTable. Summary of Comparison of Means and Correlation of Individual Body Composition Profiles With Systemic Mediators of Inflammation
eFigure 1. Comparison of Mean Inflammatory Mediator Levels in Patients Who Developed a Postoperative Complication and Those Who Did Not
eFigure 2. Comparison of Mean Inflammatory Mediator Levels in Patients Who Experienced Disease-Specific Mortality and Those Who Did Not in Patients With High Visceral to Total Fat Ratio (V:TFR)

This supplemental material has been provided by the authors to give readers additional information about their work.

© 2021 Fleming CA et al. *JAMA Network Open.*

eAppendix 1. Full Method Used to Calculate Body Composition Profiles on Axial Computed Tomography

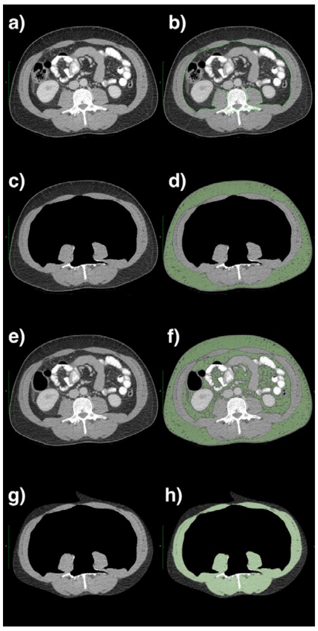

Visceral adipose area measured on a single axial CT slice 6cm above the L4-5 intervertebral disc correlates strongly with overall visceral adipose volume[63]. Total adipose tissue area and subcutaneous adipose tissue area were measured on CT slices at this location and visceral adipose area was calculated by subtraction. Skeletal muscle area measured at the L3 level is also an accurate surrogate for total skeletal muscle mass[44,64]. This muscle area is then used to define a skeletal muscle index (usually calculated as area relative to patient height (cm2/m2)), which is then used to assess patients for sarcopenia. For this study, fat and muscle volume measurements were performed using the segmentation tool in the DICOM image viewer Horos (Horos Project, 2015)[43]. The process for measuring the fat and muscle areas was as follows:

1. Define CT slice 6cm above the L4/5 intervertebral disc (**Figure 1.a**)
2. Manually outline the inside of the abdominal wall (**Figure 1.b**)
3. Remove the intra-abdominal contents form the image (**Figure 1.c**)

© 2021 Fleming CA et al. *JAMA Network Open.*

4. Define the subcutaneous fat using the segmentation tool and Hounsfield Unit limits of -190HU to -30HU (**Figure 1.d**)[65]

5. Replace the intra-abdominal contents and manually remove the faeces from the image (**Figure 1.e**) and define the total fat (visceral and subcutaneous using the same Hounsfield Unit limits (**Figure 1.f**)

6. Define the CT slice at the L3 transverse processes, remove the intra-abdominal contents, spine and skin from the image (**Figure 1.g**) and define the muscle area using Hounsfield Unit limits of -30HU to 110HU (**Figure 1.h**)[64]

© 2021 Fleming CA et al. *JAMA Network Open.*

eAppendix 2. Sex-Specific Cutoff Values for Reference Range and Pathological Body Composition Parameter Subgroups

V:TFR=visceral to total fat ratio; SC:TFR=subcutaneous to total fat ratio.

	**Male**	**Female**
**High Total Fat Area** (cm^2)	>495	>392
**High Visceral Fat** (cm^2)	>163.8	>80.1
**High Subcutaneous Fat** (cm^2)	>210	>274.6
**High V:TFR**	>0.397	>0.330
**High SC:TFR**	>0.469	>0.715
**Low SMA** (cm^2)	<141.6	<93

© 2021 Fleming CA et al. *JAMA Network Open.*

eTable. Summary of Comparison of Means and Correlation of Individual Body Composition Profiles With Systemic Mediators of Inflammation

	**Comparison of Mean BCP levels [mean (SD)]**											
	**WCC**	**CRP**	**Albumin**	**IL-1b**	**IL-2**	**IL-6**	**IL-10**	**IFNg**	**TNFa**	**VEGF**	**CD11b**	**CD14**
**Skeletal Muscle Area** (SMA, cm^2)	p=0.75	p=0.982	**p<0.001 ***	**p=0.011***	p=0.192	**p=0.019***	p=0.384	p=0.240	p=0.055	p=0.164	p=0.212	p=0.106
Normal	7.16 (2.26)	20.70 (23.18)	46.50 (0.71)	1.18 (0.15)	1.31 (0.29)	2.64 (2.90)	9.36 (1.74)	6.23 (1.05)	4.48 (1.94)	249.71 (153.29)	293.82 (36.60)	395.69 (67.8)
Low	6.87 (2.66)	24.45 (23.4)	41.19 (4.75)	1.05 (0.03)	1.21(0.17)	1.10 (0.13)	0.45 (0.23)	2.51 (1.64)	3.25 (0.65)	433.1 (34.49)	145.90 (119.9)	322.07 (131.95)
**Total Fat Area** (TFA, cm^2)	**p=0.030***	p=0.089	p=0.303	p=0.051	p=0.656	p=0.089	**p=0.012***	p=0.314	p=0.097	p=0.189	p=0.305	p=0.832
Normal	6.71 (1.44)	6.19 (4.04)	41.68 (4.15)	0.10 (0.13)	0.30 (0.31)	1.77 (1.56)	20.26 (4.34)	4.79 (6.89)	3.93 (1.50)	322.64 (275.19)	356.70 (395.97)	426.77 (378.26)
High	7.75 (3.03)	28.14 (15.26)	40.8 (5.81)	0.24 (0.12)	0.33 (0.29)	3.58 (3.67)	11.31 (3.37)	7.18 (11.18)	5.19 (2.23)	508.55 (360.70)	218.03 (201.57)	392.64 (354.99)
**Visceral Fat Area** (VFA, cm^2)	p=0.427	p=0.777	p=0.472	**p=0.019***	p=0.738	**p=0.038***	p=0.416	p=0.292	p=0.358	**p=0.001***	p=0.420	p=0.472
Normal	6.53 (1.85)	7.63 (1.34)	40 (5.10)	0.05 (0.04)	0.33 (0.30)	1.87 (1.49)	19.50 (5.88)	8.22 (10.97)	3.78 (1.54)	179.634 (20.73)	218.625 (254.59)	400.74 (378.44)
High	7.28 (2.31)	20.7 (43.18)	41.79 (4.81)	0.18 (0.15)	0.32 (0.30)	3.58 (3.93)	7.08 (2.37)	4.76 (7.56)	4.57 (1.95)	445.36 (332.9)	308.04 (338.83)	415.49 (367.89)
**Subcutaneous Fat Area** (SCFA, cm^2)	p=0.082	p=0.064	p=0.180	p=0.172	p=0.120	p=0.117	**p=0.005***	p=0.201	p=0.129	p=0.663	p=0.106	p=0.180
Normal	7.05 (1.50)	29.84 (6.60)	42 (5.10)	0.11 (0.13)	0.25 (0.26)	1.46 (1.16)	9.33 (3.3)	4.44 (6.78)	3.97 (1.45)	381.63 (290.78)	324.73 (387.79)	426.78 (381.73)
High	7.24 (3.22)	11 (3.88)	39.78 (4.10)	0.22 (0.15)	0.46 (0.33)	2.74 (2.93)	18.83 (4.72)	8.52 (11.66)	5.13 (2.33)	442.84 (374.51)	248.91 (214.43)	392.63 (349.37)
**Visceral to Total Fat Ratio** (V:TFR)	p=0.192	p=0.231	p=0.367	p=0.050	p=0.244	**p=0.008***	**p=0.002***	p=0.444	p=0.076	**p=0.013***	p=0.237	p=0.413
Normal	6.51 (0.12)	6.81 (4.01)	41.5 (5.23)	0.67 (0.63)	0.41 (0.33)	1.45 (0.95)	20.82 (4.69)	6.97 (10.24)	4.59 (1.88)	244.70 (95.17)	379.56 (106.64)	506.38 (130.51)
High	7.33 (2.46)	23.56 (4.12)	41.17 (3.7)	1.74 (1.55)	0.28 (0.28)	3.23 (3.30)	10.90 (3.30)	5.02 (7.75)	4.35 (1.93)	514.03 (371.96)	264.33 (98.61)	377.72 (39.84)
**Subcutaneous to Total Fat Ratio** (SC:TFR)	p=0.168	p=0.236	p=0.085	p=0.298	p=0.326	p=0.055	**p=0.044***	p=0.685	p=0.188	p=0.281	p=0.282	p=0.249

© 2021 Fleming CA et al. *JAMA Network Open.*

Normal	7.54 (2.57)	22.36 (4.88)	40.64 (5.58)	0.19 (0.14)	0.37 (0.30)	2.60 (2.98)	4.39 (4.24)	4.49 (8.76)	4.84 (2.20)	415.33 (24.05)	343.69 (365.07)	485.93 (380.52)
High	6.46 (1.37)	4.05 (3.66)	42.5 (3.47)	0.11 (0.14)	0.25 (0.29)	2.22 (1.98)	9.54 (8.19)	6.36 (8.07)	3.84 (1.20)	372.81 (25.10)	208.88 (232.99)	307.18 (320.90)

	**Correlation Co-Efficient (r)**<br>r (95%CI) p-value											
	**WCC**	**CRP**	**Albumin**	**IL-1b**	**IL-2**	**IL-6**	**IL-10**	**IFNg**	**TNFa**	**VEGF**	**CD11b**	**CD14**
**Skeletal Muscle Area** (SMA, cm^2)	0.190 (-0.198-0.535) 0.334	-0.004 (-0.554-0.549) 0.991	0.028 (-0.379-0427) 0.895	0.070 (-0.425-0.533) 0.790	-0.149 (-0.537-0.291) 0.508	0.062 (-0.326-0.432) 0.760	-0.141 (-0.515-0.278) 0.512	-0.179 (-0.559-0.262) 0.425	0.031 (-0.413-0.361) 0.881	0.008 (-0.387-0.374) 0.970	-0.309 (-0.633-0.108) 0.143	-0.394 (-0.700-0.033) 0.070
**Total Fat Area** (TFA, cm^2)	0.332 (-0.047-0.627) 0.085	0.382 (-0.214-0.771) 0.197	0.061 (-0.351-0.453) 0.779	0.432 (-0.061-0.756) 0.083	-0.082 (-0.487-0.352) 0.718	0.464 (0.102-0.718) **0.015***	-0.075 (-0.465-0.339) 0.727	-0.093 (-0.495-0.342) 0.682	0.430 (0.051-0.701) **0.028***	0.249 (-0.144-0.575) 0.210	-0.262 (-0.602-0.158) 0.216	-0.097 (-0.495-0.342) 0.681
**Visceral Fat Area** (VFA, cm^2)	0.352 (-0.024-0.641) 0.066	0.547 (-0.006-0.844) 0.053	0.138 (-0.281-0.513) 0.521	0.341 (-0.167-0.706) 0.181	-0.162 (-0.546-0.279) 0.472	0.356 (-0.028-0.648) 0.069	-0.160 (-0.529-0.260) 0.455	-0.187 (-0.564-0.255) 0.404	0.434 (0.055-0.703) **0.027***	0.239 (-0.155-0.567) 0.231	-0.247 (-0.591-0.174) 0.245	-0.105 (-0.505-0.331) 0.641
**Subcutaneous Fat Area** (SCFA, cm^2)	0.076 (-0.306-0.437) 0.700	-0.353 (-0.757-0.246) 0.236	0.141 (-0.515-0.278) 0.512	0.337 (-0.172-0.704) 0.186	0.147 (-0.293-0.535) 0.514	0.376 (-0.005-0.661) 0.053	0.152 (-0.268-0.523) 0.478	0.170 (-0.271-0.552) 0.450	0.304 (-0.094-0.619) 0.131	0.105 (-0.287-0.466) 0.603	-0.087 (-0.474-0.328) 0.687	0.007 (-0.416-0.428) 0.975
**Visceral to Total Fat Ratio** (V:TFR)	0.259 (-0.126-0.576) 0.183	0.475 (-0.104-0.813) 0.101	0.161 (-0.259-0.530) 0.452	0.208 (-0.303-0.626) 0.423	-0.189 (-0.565-0.253) 0.400	0.107 (-0.285-0.468) 0.595	-0.283 (-0.616-0.136) 0.181	-0.335 (-0.663-0.101) 0.128	0.024 (-0.408-0.367) 0.908	0.133 (-0.260-0.488) 0.509	-0.209 (-0.565-0.212) 0.327	-0.192 (-0.567-0.250) 0.393
**Subcutaneous to Total Fat Ratio** (SC:TFR)	-0.271 (-0.585-0.114) 0.164	-0.175 (-0.475-0.104) 0.201	-0.160 (-0.530-0.260) 0.454	-0.218 (-0.632-0.293) 0.400	0.196 (-0.246-0.570) 0.383	-0.115 (-0.474-0.277) 0.568	0.281 (-0.138-0.615) 0.183	0.333 (-0.103-0.662) 0.130	0.015 (-0.375-0.400) 0.943	-0.144 (-0.497-0.249) 0.473	0.208 (-0.213-0.564) 0.330	0.190 (-0.252-0.566) 0.397

**Linear association**
**Inverse association**
***p<0.05**

© 2021 Fleming CA et al. *JAMA Network Open.*

eFigure 1. Comparison of Mean Inflammatory Mediator Levels in Patients Who Developed a Postoperative Complication and Those Who Did Not

A. low skeletal mass area (SMA) and B. high visceral:total fat area (V:TFR).

*p<0.05; **p<0.01; ***p<0.001.

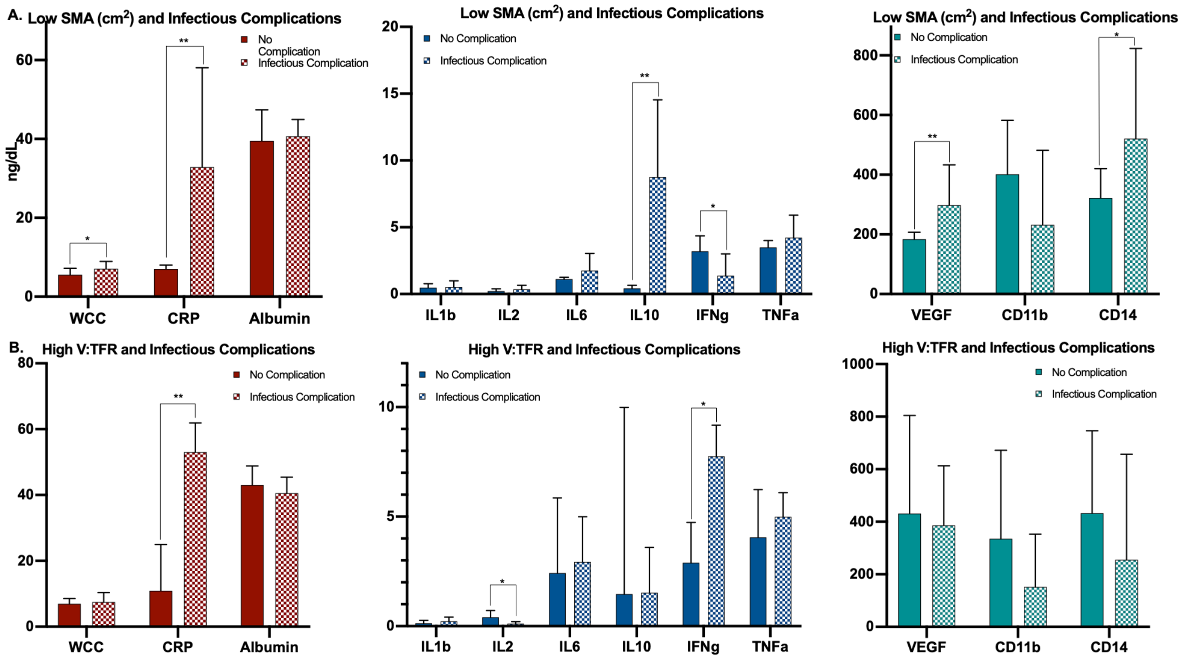

© 2021 Fleming CA et al. *JAMA Network Open.*

eFigure 2. Comparison of Mean Inflammatory Mediator Levels in Patients Who Experienced Disease-Specific Mortality and Those Who Did Not in Patients With High Visceral to Total Fat Ratio (V:TFR)

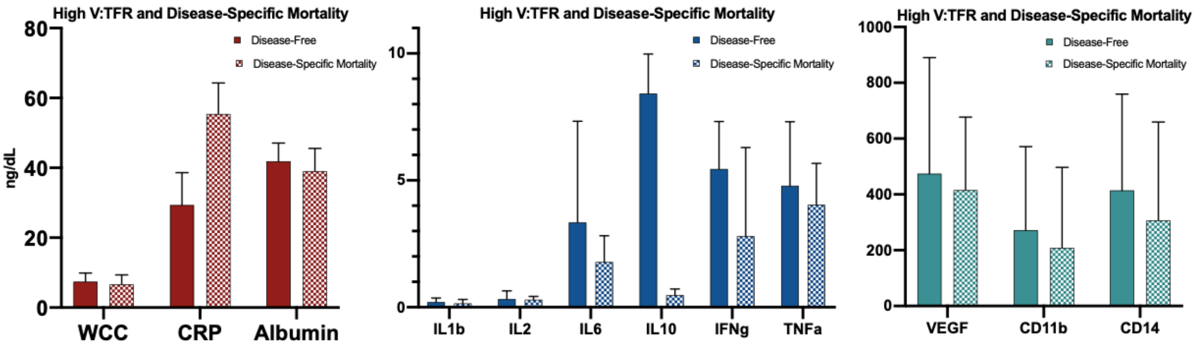

© 2021 Fleming CA et al. *JAMA Network Open.*
